# From e-Health to i-Health: Prospective Reflexions on the Use of Intelligent Systems in Mental Health Care

**DOI:** 10.3390/brainsci8060098

**Published:** 2018-05-31

**Authors:** Xavier Briffault, Margot Morgiève, Philippe Courtet

**Affiliations:** 1 Centre de Recherche Médecine, Sciences, Santé, Santé Mentale, Société (CERMES3), UMR CNRS 8211-Unité Inserm 988-EHESS-Université Paris Descartes, 75006 Paris, France; xavier.briffault@parisdescartes.fr; 2 FondaMental Foundation, 94000 Créteil, France; philippe.courtet@univ-montp1.fr; 3 Institut National de la Santé et de la Recherche Médicale U1061 Neuropsychiatry: Epidemiological and Clinical Research, University of Montpellier, 34000 Montpellier, France; 4 Department of Psychiatric Emergency & Acute Care, Lapeyronie Hospital, CHU Montpellier, 34000 Montpellier, France

**Keywords:** m-health, i-health, depression, nosography, categorizations, symptoms networks, ecological momentary assessment, ecological momentary intervention, fictional case study

## Abstract

Depressive disorders cover a set of disabling problems, often chronic or recurrent. They are characterized by a high level of psychiatric and somatic comorbidities and represent an important public health problem. To date, therapeutic solutions remain unsatisfactory. For some researchers, this is a sign of decisive paradigmatic failure due to the way in which disorders are conceptualized. They hypothesize that the symptoms of a categorical disorder, or of different comorbid disorders, can be interwoven in chains of interdependencies on different elements, of which it would be possible to act independently and synergistically to influence the functioning of the symptom system, rather than limiting oneself to targeting a hypothetical single underlying cause. New connected technologies make it possible to invent new observation and intervention tools allowing better phenotypic characterization of disorders and their evolution, that fit particularly well into this new “symptoms network” paradigm. Synergies are possible and desirable between these technological and epistemological innovations and can possibly help to solve some of the difficult problems people with mental disorders face in their everyday life, as we will show through a fictional case study exploring the possibilities of connected technologies in mental disorders in the near future.

## 1. Introduction

### 1.1. Context

The notion of “depressive disorders” covers a set of disabling problems, often chronic or recurrent, whose prevalence in the general population is high. They are generally characterized by a high level of psychiatric and somatic comorbidities and a high proportion of chronicity or relapses. For these reasons, they represent an important public health problem and considerable suffering for the people who endure them and their families. To date, therapeutic solutions remain unsatisfactory, despite the intensity of research on this subject for decades. Many researchers today consider that this lack of satisfactory results is only temporary and that decisive therapeutic innovations may emerge in the future within the current paradigm, despite the fact that such results have not been obtained in several decades. Others, in contrast, see it as a sign of a decisive problem in the paradigm in which disorders are conceptualized. One of the central problems is that, although the particularly multifactorial, “bio-psycho-social” nature of these disorders—like many other mental disorders—is the subject of international consensus, it remains classic, however, to consider these multiple factors only as contributing to the development of an underlying cause of an isolable disease, the symptoms of which would only be the observable effect of that cause. The various symptoms of depression would thus be the effect of “depression”, just as the multiple symptoms of syphilis are the effect of pale treponemal infection, which is the sole initial cause. 

This organizational scheme is particularly visible in the Diagnostic and Statistical Manual of Mental Disorders (DSM) [1]. This reference nosography of the American Psychiatric association (APA), which has been organizing international research on mental disorders for several decades, adopted in its third version (DSM-III, published in the early 1980s) a categorical definition of disorders independent of their context of occurrence. Disorders are constructed by composition of elementary observable symptoms not articulated with each other or with context to obtain a polythetic definition (i.e., composed of a “mandatory” core of symptoms to which are added “optional” symptoms). This syndrome is supposed to represent the theoretical level immediately above the elementary symptoms, each definition being the supposedly reliable formalization (i.e., of a high degree of intersubjective agreement) of phenomenal random variations statistically observed around a supposed natural morbid entity which would be the valid denotation [2,3]. 

This approach to mental disorders is more generally part of a “medical model” of disorders [4]. In this medical model the patient is thought of under the angle of a specific problem that he has, and which can be circumscribed and isolated, this problem being directly caused by one (at most some) underlying cause(s), these causes directly explaining the problem. This conceptualization directs research towards the hypothesis that there are therapeutic ingredients specific to each of these causes. Each of these ingredients having a specific efficacy that must be evaluated by randomized controlled trials specifically constructed to evaluate the intrinsic efficacy of an intervention independently of any context. For several decades, this approach has produced an important list of empirically supported treatments (i.e., for which a statistically significant positive effect size has been calculated in randomized controlled trials), whether pharmacological, psychotherapeutic, neurosurgical, and so on, treatments described as precisely as possible in protocol manuals defined independently of the context. While it is undeniable that these multiple treatments have therapeutic efficacy, the fact remains that this efficacy is still low and that many disorders and patients are not or only slightly improved by these approaches [5,6]. 

The normative ideal of an isolable mental disorder with a single cause massively organizes the collective functioning of research and practice on mental disorders. It prevents significant interactions between symptoms from being taken into consideration and syndromic entities used to characterize mental disorders from being considered as something other than the manifestations of the natural entity that is supposed to cause them. It is also massively structuring possible therapeutic practices, because it prohibits the hypothesis that the symptoms of a categorical disorder, or of different comorbid disorders, can be interwoven in chains of interdependencies. It thus prevents to conceive intervention acting independently and synergistically on the elements of theses chains to influence the functioning of the symptom system, rather than limiting oneself to targeting a hypothetical single underlying cause. 

This new paradigm, thought still in its infancy and under debate, appears as an interesting framework for conceptualizing mental disorders, particularly depressive disorders. If we adopt this paradigm, there is an important need to rethink the conceptual bases of physio-psycho-socio-pathological knowledge that underlie the conceptions of possible therapeutic approaches, and to invent new observation tools allowing a better phenotypic characterization of disorders and their evolution.

### 1.2. Objectives

The objective of this article is to propose some elements for reflection on possible synergies between recent developments in the conceptualization of mental disorders, particularly depressive disorders and innovations that are developing very fast in the field of connected objects and applications commonly grouped under the terms e-health/m-health. More precisely, we propose a systematised yet speculative use case of these new technologies based on a fictional case study of a person presenting complex mental health problems. Our main goal with this case is to propose a concrete example of how intelligent connected information systems could be useful in clinical and therapeutical settings in order to ecologically augment clinical observations and therapeutical interventions in mental health. Moreover, while empirical analysis of the current uses of Ecological Momentary Assessment (EMA)/ Ecological Momentary Intervention (EMI) devices is essential, the conception of these devices is still limited by conceptual limitations that prevents such empirical analyses to serve as a basis for an in-depth improvement of such tools. Consequently, it is also necessary to anticipate the upheavals that will occur in the near future by reflecting on possible, but not yet proven, uses of these devices for people with psychological problems/mental disorders, long before it can, eventually if proven safe and useful, be proposed in clinical routines.

## 2. New Approaches in Conceptualizing Mental Disorders

A current of research whose foundations can be traced back to the end of the 2000s [7,8] and which has largely developed since then [3,9] is fully in line with the paradigmatic rupture that we evoke. It proposes a new conceptual and therapeutic approach that makes it possible to unessentialize mental disorders by redefining them as clusters of properties connected between them by a homeostasic system of causal relations, thus going over the limits of the DSM that we have just mentioned. The disorders are then conceived as stable attractors in a network of properties, which emerge from the dynamic organization of causal links within the network rather than being arbitrarily reified into independent static entities. This type of networked approach allows for the adoption of “a psychosystemic approach that addresses the inherent complexity of mental disorders by using explicit models of the interactions between their psychological, biological and social characteristics that play a role in the development of psychiatric conditions, understood as clusters of causally related properties” [10]. As a result, the first category of apprehension of persons with mental disorders is no longer limited to the isolated, monadic individual suffering from an isolated disease caused by a single cause. Instead, today it tends to evolve towards an agent in situation whose action is disturbed by inadequacies between his characteristics and those of his situation, characterizable in real time by multiple parameters in mutual interactions articulated to the parameters of the situation.

In addition to the “internal” causal relationships linking the symptoms of a given psychiatric disorder together, these new approaches also make it possible to integrate the dynamic couplings between the modes of functioning internal to the individual and those that organize social, relational, physical environments and situations… of which he is part, with a specific interest in the “depressogenic”, “anxiogenic”, “OCDgenic”, “autistogenic”, “suicidogenic”, “disadaptogenic”, “burn-outogenic”… properties of these couplings. The pathogenic or disabling dimension here no longer belongs solely to the individual or solely to the situation but to the joints of both. This logic no longer looks only at the causes, but also at the consequences in a given environment of health problems in order to classify them [11]. In this shift from the patient with a psychiatric-neurological illness to the “person in situation”, the medical objective of reducing or eliminating symptoms loses its centrality. It becomes one of the elements of an approach aimed at reducing the daily impact of these symptoms in order to improve the quality of life [12,13,14]. A shift is thus taking place from a strictly curative logic inscribed in a medical model to a rehabilitation logic also inscribed in a contextual model. The therapeutic approach aims at the reduction of functional consequences only by the reduction/suppression of the pathology. The new situated approaches additionally open the possibility of acting directly on the disabling consequences in ecological situation, not only by looking at the “big” effects of the “big” pathological entities, but also and above all by looking at the articulations of individual micro-mechanisms and socio-environmental micro-mechanisms in order to be able to intervene finely on them.

These conceptual developments in mental health are thus no longer based solely on large diagnostic entities (depression, schizophrenia, etc.) but on pluralities of local dysfunctions or problematic stabilizations in graphs of interactions of “symptoms” inscribed in a process [15,16,17]. The focus is no longer limited to the single “person” pole but extends to its functioning in a real environment [18]. They make it possible to imagine new forms of action that are finer, more contextualized, more ecological, more interactive, more focused on changes in interactions between the various components of the person and the various components of his or her real environment.

## 3. New Opportunities for Observation

These innovations in the conceptualization of mental disorders open up new clinical and therapeutic possibilities. It is now theoretically possible not only to observe with much finer granularity the components of a mental disorder and their internal interactions with the components of the situation and the environment, but also to intervene finely on these components and on the causal chains that maintain the system in patterns that are pejorative for the person. To these new theoretical possibilities, different emerging technologies offer operational implementation possibilities.

This is particularly the case for connected mobile technologies that are developing in the e-health/m-health field. Connected technologies, particularly because of their very small size and ubiquitous communication capabilities, offer hitherto completely unprecedented possibilities for collecting multiple data in real time [19,20,21] associated with a space-time location on a person and her proximal or distal environment [22], but also to intervene at multiple levels on this person and this environment by coupling in real time the interventions to the collected data. To date, no other therapy, care or support system has had such an ability to integrate into the subject’s life in order to gather information and intervene, whether it is the classic office consultation, home visits, telemedicine, or even complete hospitalization, which certainly allows total observation of the patient, but has nothing ecological about it. By making possible the simultaneous use, in real time and in ecological situations, of multiple micro-sensors, micro-effectors and human actors, all interconnected, the new connected technological devices offer unprecedented possibilities for reducing the grain of data collection and interventions on the person. The fineness of the possible articulations of individual parameters with each other and with multiple situational parameters, as well as the spatial and temporal perimeter of the accessible information [23], is thereby massively increased.

## 4. Synergies between Conceptual and Technological Innovations

This is why the epistemological evolutions we have just mentioned-“network” approaches to mental disorders-and the evolutions of connected technologies in the field of health (e-health, m-health)-objects connected in networks-tend today (although obviously coming from different epistemic universes) to join. It allow in mental health problems micro-interventions much more targeted, evolutive, and contextualized than allowed by the previous approaches applying “macro-interventions” to “macro-pathologies” [24]. The patient is no longer seen as a monadic individual altered by diseases, occasionally encountered out of context by practitioners who have only clinical and paraclinical observations, supplemented by the patient’s own clinical and paraclinical observations, accessible in the limited space of the consultation for any information on the patient’s functioning. Instead, the patient tends today to evolve towards an agent in a situation whose action is disturbed by inadequacies between his functions and those of his situation, characterizable in real time by multiple parameters in mutual interactions articulated to the parameters of the situation [16,17,25], on which multiple people using multiple technical devices (applications and connected objects) can now intervene in real time and in an ecological environment.

We now have the ability to generate in real time a “cloud of data” associated with the person and his situation, to statistically analyze co-variations in order to produce inferences based on “data-based” or “data-driven” predictive models. We are also able to integrate these data directly into a priori models (when available) of the relationships between the measured variables, in order to produce “model-based” predictions, and to make all these elements available to the patient and his therapists, in the service of an “augmented” clinic and therapy [26]. Among these multiple parameters whose measurement is now possible, some can be considered as items on which we wish to act specifically, either directly, or through causal factors, or assumed to be causal on the basis of strong statistical correlations derived from analysis of groups or intra-individual correlations. In the case of mental disorders, one can for example wish to act on mood, anxiety, fear, stress, sleep, arousal, motivation, sedentary life, mental suffering, aversive tensions, suicidal ideations, obsessions, compulsions, consumption of psychotropic substances, eating habits, social relations, hallucinations, executive functions, the presence to oneself and to the environment, muscular tensions, memory, reasoning, somatoform symptoms [27,28,29,30,31,32]. In short, on any psychological, physiological, behavioural parameter… considered problematic and within the scope of “mental health”, and for which there would be a direct means of action or an upstream influencing factor. A considerable number of applications have been developed in the field of e-mental health, to the point that it can almost be said that whatever the problem is considered “there is an application for that”, according to the title of a recent article [33]. This is particularly the case for depressive disorders [29,34]. While most of these applications are not evaluated, available results show that these technologies can be not only effective but also cost-effective [35,36,37].

## 5. From e-Mental Health to i-Mental Health

However, these applications generally continue to fall within the “isolable disorder/specific intervention” paradigm, the limits of which have been shown and do not allow the best use to be made of isomorphism between a network approach to disorders and a network approach to connected objects. There is therefore reason to fear that the phenomenon of conceptual limitation observed in the design of past therapies may be repeated in the future for these new technologies if conceptual frameworks do not evolve sufficiently. This is the hypothesis put forward by various authors, including Berrouiguet et al. [38], who call for the “e-health” paradigm to be overcome by a broader approach they call “i-health” (intelligent health), or Briffault et al. who introduce the concept of the Technologically Augmented Clinical and Therapeutic relationship (TACT) [24,39,40].

According to these authors, the usual approach to psychiatric care is limited in its possibilities of data collection, diagnosis and intervention by the very characteristics of consultations between practitioners and patients, brief, rare, taking place outside any real life context and in which relatives rarely participate. The tools currently proposed in the field of e-health certainly make it possible to extend the collection of data during the periods between consultations, by Internet or by mobile phone and to store them in personal electronic files. But this collection remains however more often than not punctual, it requires an intervention of the patient, does not allow the collection in real time of personal and contextual information. In truth, it does not qualitatively improve the possibilities of medical analysis.

Now, what connected objects and the applications that accompany them offer today is the possibility of interconnecting in real time the multiple components of the person in action. These technologies offer the possibility of articulating: vertically the macro (social, smart cities…), meso (situation, smart homes, physical and relational environment) and micro (the person) and even very micro (different physiological and psychological parameters and mechanisms) levels; and horizontally the different domains of existence (nutrition, activities, relationships, movements, organization, cognitions, affects…) involved in daily living. All elements whose proper functioning and articulation are disturbed by mental disorders and can contribute to their persistence or reduction.

These new devices thus offer new possibilities for spatial, temporal, and thematic extensions of psychiatric, psychotherapeutic, psycho-educational, medical relationships…, whose observation and intervention potential can now be extended far beyond consultation to potentially concern all areas of life, at any time and in any place, while integrating the possibilities for automatic analysis and analysis assistance offered by data-mining and deep-learning technologies. As Berrouiguet et al. point out, this is the beginning of a twofold evolution [38]. First of all, the routine integration of the data collected in the psychiatric consultation situation will give practitioners real-time access to subjective and behavioural data from patients and their families, which will significantly modify their clinical observation and therapeutic intervention possibilities. Then, the addition to the “handmade” analysis of the data by the practitioner-made impossible by the amount of data generated-of automatic analysis possibilities will allow the development of intelligent medical decision support systems allowing the development of predictive models and personalized treatments.

This is no longer only a quantitative evolution, but a real qualitative revolution. From the cylinder-rolled sheets of Laennec’s first stethoscope to the most sophisticated MRIs or biological analyses, and even current Ecological Momentary Assessment applications, there is no real qualitative leap. Tools of “mediate auscultation”, these technologies only improve its possibilities, without modifying its basis: it is always the physician who makes sense of the observed data, in the clinical context integrated into his relationship with the patient. Never has a scanner, a sheet of biological results, a psychiatric evaluation scale or its smart phone version attempted to analyze by themselves what they were giving the doctor as data on the patient’s condition. But this is what connected technologies combined with artificial intelligence technologies allow today, which justifies their being considered “disruptive”. Indeed, they lead to a break in continuity in the status of observation and intervention tools in medicine and mental medicine; they move them from the stage of tools for extending the possibilities of the doctor to the stage of tools capable of proposing interpretations or even making decisions and carrying out actions themselves, in interaction with the various stakeholders in a care process (people with disorders, mental health practitioners, relatives, etc.).

## 6. What Elements for an i-Mental-Health System

To provide the best possible service to people with mental health disorders, the i-mental-health devices of the future will need to build on both the technological and conceptual innovations we have presented. They should take note of the fact that the health parameters involved in the functioning of a person in a situation are multiple and diverse, that they are in complex and dynamic interactions, that these interactions concern parameters belonging to different levels (genetic biological, psychological, situational, contextual, social) that influence each other inter and intra-level, and that the system disturbances that constitute the relationships between these health parameters have functional consequences that depend on the individual in the situation. The value attributed to them is also individual and may vary according to times and situations within the same individual. The network of interactions between health parameters can evolve very gradually or very suddenly towards states with pejorative functional consequences, or even towards morbid states that stabilize and can be considered as characterized disorders [17,26,41].

An i-mental health system must offer its users (professionals, patients, relatives) a set of health parameters, enable them to select those they wish to monitor according to their characteristics and their situation. It must also provide them with the means to highlight the fine relationships between these health parameters in order to be able to determine relevant places and means of intervention, based as much on the possibilities of automatic data-based and model-based analyses as on the possibilities of a clinical augmentation that these new tools/conceptualizations offer to users.

## 7. What Observation Tools

Any health parameter selected as a relevant observable must be capable of being monitored by an adapted observation tool. In order to increase compliance and to preserve the ecological validity of the data collection, the use of tools must be as simple and transparent as possible and be able to fit into the flow of activity without disrupting it. For example, in all possible cases, objective passive sensors that do not require any user action should be preferred to the use of questionnaires that consume time and disrupt the flow of activity, and that are also subject to recall bias and various cognitive and desirability biases.

Nevertheless, the use of questionnaires remains unavoidable in certain cases, typically for parameters related to user subjectivity. For example, no passive sensor can measure the degree of psychological suffering, assess quality of life, evaluate the level of aversive tension or the feeling of having a meaningful life, and so on. At the other end of the spectrum, no subjective assessment can better than an objective measure account for the degree of activity (actimeter), heart rate (heart rate monitor), blood pressure (blood pressure monitor), sleep characteristics (actimeter + heart rate monitor + other specific sensors of brain activity), biological markers, and so on.

However, the subjective consequences of variations in an objective parameter remain within the user’s appreciation and expression. As a simple example, a tennis player and a footballer will not give the same value to a wrist tendonitis, even if the pathology is “the same”. Some parameters also have intermediate statuses in that they can be observed by different complementary means. An example is depression. If it can be evaluated by self-administered or hetero-administered standardized questionnaires (Hamilton scale [42], Beck scale [43], PHQ-9 [44]…), simplified subjective assessments (visual analogue scales of depressive feelings [45]), it is also clinically - and therefore more or less objectively - observable, whether it is by clinical collection by a trained professional, or proxies whose predictive value is more or less good (different parameters of voice, activity, motor skills, relationships, lexical and semantic content of communications, etc.) [46,47,48,49]. A conjunction of observation instruments therefore makes it possible to triangulate the observed value for this type of parameter.

## 8. Process for Designing and Using an i-Mental-Health Device

The development of i-mental health systems integrating the functionalities that have been presented is a complex process, which is beginning to be the subject of new work [50,51,52]. An integrated device will necessarily have to rely on a team of health professionals trained in the use of connected devices, able to propose a set of functionalities adapted to each user according to his needs and situation, to analyse the data collected and to propose interventions. These professionals will need to be able to rely on a platform that allows them to generate an integrated suite of mobile applications in a totally flexible way to the patient/therapist’s choice by integrating pre-existing or custom-developed functionalities that can be reprogrammed according to the patient’s evolution. This suite should be capable of: (a) collecting health data in programmable ways; (b) using data from diverse eHealth/wellness applications; (c) aggregating standardized sensor flows and commercial applications; (d) providing therapeutic and prevention support and accompaniment tools; (e) dialogue with environmental servers (smart cities, smart homes, etc.); and (f) proposing user interfaces allowing data sharing for co-analysis and co-decision purposes between users, healthcare professionals, relatives, other stakeholders, and so on.

Co-decision on the choice of observation and co-design tools for the intervention programme involves the patient and a multidisciplinary team of health professionals, specially trained in the use of connected health tools in a consultation (which can be carried out remotely). In collaboration with the patient, this involves formalizing the main relevant elements of his health problems and situation in order to set up a complete observation and intervention application based on health parameters, questionnaires, connected objects and interventions available within the device. In order for the consultation to be as effective as possible, rapid training in the principles of prevention/promotion of health that will be used can be offered prior to the consultation to the patient in the form of an e-training/psychoeducation tool. If there are specific needs not covered by existing modules, specific developments may be implemented. They are then genericized and integrated into the basic proposal. The patient should ideally be able to connect to the software device specifically generated for him on his various computer hardware (computer, tablet, smartphone, connected objects) as soon as the consultation is over, and follow a quick (tele)training course on its use. The patient then lives his life using the different applications, connected objects, questionnaires that are proposed to him, analyses his data using the graphic tools made available to him, and implements the actions that are proposed to him by his device. He may contact the multidisciplinary team at his own initiative, or on the proposal of the system, to examine specific health problems highlighted by the use of the device, which may lead to changes in the device (new observations, new interventions). At the same time, the health team can contact the patient, based on a human and/or automated analysis of his data, in order to propose new interventions.

The data collected by the observation devices and the interventions implemented are stored in the database, thus allowing individual real-time, localized longitudinal monitoring. Data aggregation for all users provides a representation of user health parameters and behaviors and provides objective information for inter-individual comparisons and quasi-experimental evaluations.

## 9. A Fictional Case Study

**Figure 1:**
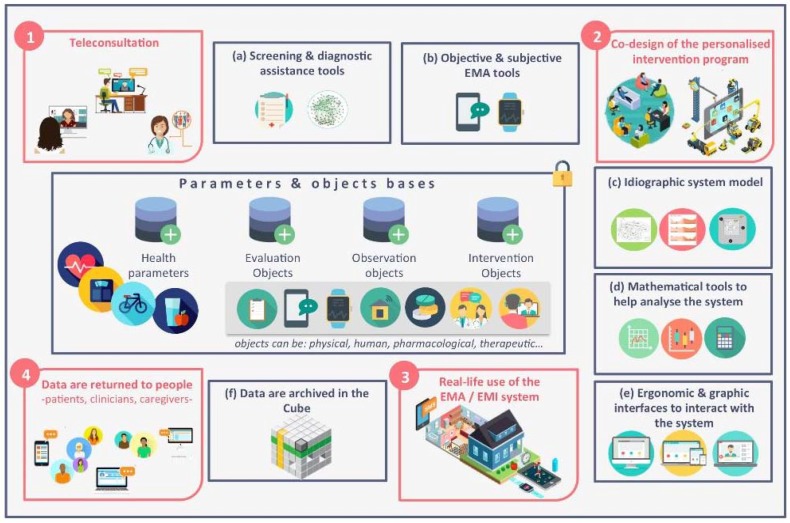
Design and use processes of a device for a Technologically Augmented Clinical and Therapeutic relationship.

**Figure 2:**
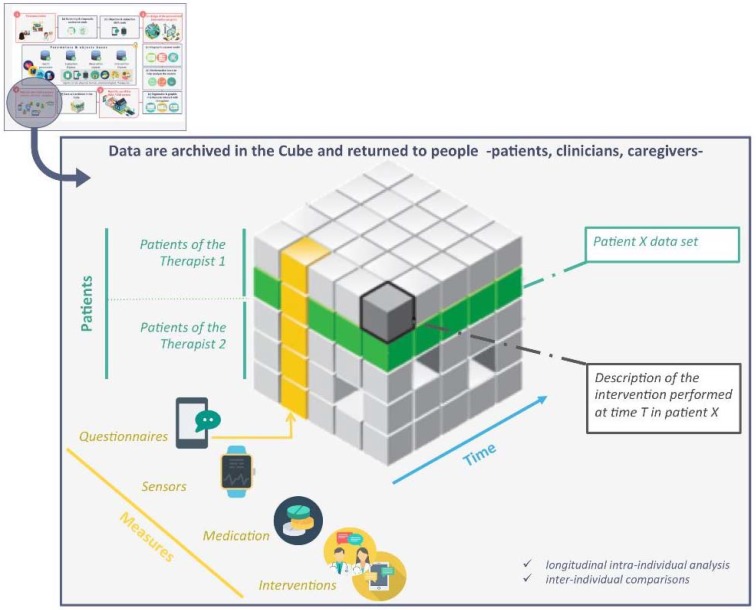
Patients X measures X time database.

The (mental) e-health field is particularly active, producing new devices at an extremely rapid pace. While empirical analysis of the current uses of these devices is essential, it is also necessary to anticipate the upheavals that will occur in the near future by reflecting on possible, but not yet proven, uses of these devices for people with psychological problems/mental disorders. Fictive case analysis offers such opportunities to reflect in advance on emerging technologies whose “disruptive” potential requires that reflections are not limited to known uses. This is what we propose here with a constructed case of depressive problem in which we imagine the possible design and use processes of a device for a Technologically Augmented Clinical and Therapeutic relationship (TACT), (Figure 1 and Figure 2).

Bob is a married man in his fifties, with two children, working on a stable job in an insurance company, living in a remote rural area, 90 min by car from the nearest metropolis, with few medical resources and even fewer psychological and psychiatric resources, a situation in which the use of connected devices is particularly suitable [53]. Confronted for several months with recurrent back and abdominal pain, palpitations, sleep problems and fits of profuse sweats, he consulted his general practitioner. Neither the clinical examination nor the paraclinical investigations carried out make it possible to highlight somatic causes to the symptoms of which Bob complains. His doctor then suggests that he consults a multi-professional e-mental health team to which he can give access, with his consent, to the data he has already collected. The financial coverage of the care protocol that will be put in place will be provided by Bob’s supplementary insurance, funded by his company as part of a collective psychosocial risk prevention program.

The data already collected and an initial teleconsultation interview (Figure 1, step 1) with Bob guide the team towards an initial set of hypotheses on causes related to the anxiety-depressive constellation of symptoms Bob complains about. In addition to the symptoms in the foreground that motivated the consultation with the general practitioner, the interview shows that Bob feels depressed. He cannot do even the smallest project, he does not want anything, he cannot have pleasure in anything. He has relationship difficulties that are unusual for him, can no longer get in touch with others, and feels that people are moving away from him. He feels in his work unbearable psychological pressures, feels controlled and monitored constantly, has the feeling of growing dehumanization, of being treated as a number and having to treat his clients as such. Although he thinks that it is his company that has changed and not his point of view, he also says he prefers to abandon all responsibility to content himself with subordinate tasks rather than continue to occupy a position that is nevertheless more rewarding and meaningful, but that requires the mobilization of cognitive, emotional and executive resources that he no longer seems to have. The interview also shows that Bob attributes his difficulties not only to the objective evolution of his work context, but also partly to two things that do not depend on it. On the one hand a painful event, the premature death of his mother following an illness when he was a young teenager. He considers that he has not mourned this very protective mother who, in his opinion, has not made him mentally strong enough and equipped him with the skills to face life’s difficulties on his own. Her advice, her presence, her support are missing more and more painfully with each problem encountered. Added to this is a growing sense of guilt with the feeling that his children are failing and not moving forward in life. While one is unemployed, uses cannabis and spends his time playing video games, the other has no education and has fallen into delinquency. He is desperate about their life trajectory, and experiences the failure of his attempts to help them as a personal failure and proof of his incompetence.

He says he always needs someone behind him to support him and tell him that he is on the right track. Although he feels that his situation is objectively difficult and generates suffering, he questions the fact that his psychological state can contribute to make him increase the difficulties of the situation at the expense of possible improvements.

After this first interview, a set of additional information gathering devices are proposed to Bob in preparation for a second interview. In order to refine the diagnostic elements, it is suggested that he answers a self-administered online questionnaire adapted from Structured Clinical Interview for DSM (SCID), the Screening Assessment for Guiding Evaluation-Self-Report-SAGE-SR, [54], which makes it possible to obtain elements on the connection of the symptoms described to the various syndromic categories of the DSM. The multidisciplinary team has a platform giving them access to different databases of connected objects, software databases, health parameters from which they can select what is relevant to Bob’s situation. This is how he is offered a dedicated application integrating the monitoring of his sleep, his daily activities, his heart rate, his eating habits and his emotional state in the different situations of daily life [55]. An activity sensor and a GPS in the form of a connected watch not identifiable as a medical device, and usable in everyday life makes it possible to collect objective physiological and behavioral data. A home automation sensor located in the home rooms collects environmental data (temperature, noise, light, air quality, presence in the room). An analysis of the use of its means of communication (telephone calls, sms, e-mails, social networks) is also implemented [48,56,57].

Three weeks later, the multidisciplinary team thus has a set of psychopathological data based on a validated self-evaluation instrument and objective behavioral and environmental data to supplement the first clinical and paraclinical examinations. These data can be used during a second more in-depth teleconsultation (Figure 1, step 2) designed to co-construct with Bob an idiographic model of the main somatic, psychological, relational and situational factors involved in the genesis and maintenance of his problems [26] and to propose adapted interventions. The data collected confirms the existence in Bob of several elements relating to anxious; depressive and adaptation disorders constellations; inefficient coping modalities in different professional situations and in interactions with his children; indicators of difficulties in managing different stressors (intense heart rate acceleration, strong emotional variations) in particular public speaking and interaction with customers; sleep difficulties and late night insomnia associated with intense snoring episodes and breathing interruptions suggestive of sleep apnea; as well as an excessively low level of physical activity and sedentary lifestyle associated with a limited scope of life, with no outside activity [58]. Diet monitoring shows a very unbalanced diet, with an excessive load of high glycaemic index foods, too little protein and excessive alcohol consumption, especially when returning home and in the late evening. As for the analysis of communications, it shows that Bob has cut himself off from his social and friendly network, and that his communications are limited to exchanges with his wife, most often associated with episodes of anxiety during his travels and professional activities. Automated analysis of his text messages shows significant use of a lexical field associated with anxiety, exhaustion and panic [59], while automatic analysis of his voice during communications also shows anxious elements in communications with his wife, and elements of depression and emotional restriction in his exchanges with clients and colleagues [60].

During this second consultation, the multidisciplinary team and Bob can study together the different intervention modalities available in the system bases in order to choose those that seem best adapted to Bob’s needs and current functioning. This is why a tele-psychotherapy is proposed to him in order to address what he has identified as one of the important causes in his current difficulties, namely the badly managed mourning of his mother’s death. Added to this are various software modules designed to enable him to better manage his insomnia problems (e.g., Sleepcare [61]), to gradually overcome his difficulties in making projects, his aboulia and his anhedonia thanks to e-CBT protocols (e.g., MoodHacker [62] and Get Happy [63]), improve psychological flexibility, regulate emotions and guilt more effectively, and identify what is really important to him in life in order to act in this direction using elements of Acceptance-Based Therapy (e.g., ACTsmart [64] and ibobbly [65]), to facilitate his organization and improve his attentional control, especially at work in order to help him better cope with psychological pressure problems (e.g., LivingSMART [66]), to support his sense of self-esteem, acceptance of his personal identity, in order to improve his self-image, especially in relationships with others, particularly with his children, customers and superiors (e.g., SuperBetter [67]). Bob’s sleep difficulties are the subject of several synergistic interventions. The connection of the e-mental health device to home automation devices allows a dynamic management of the environment adapted to the sleep profile. The system thus acts on the inside home temperature, progressively reduces the intensity of light and the quantity of blue light diffused by the screens and the connected bulbs, proposes adapted relaxation and sleep preparation interventions at appropriate times, and suggests an appropriate distribution of food categories for dinner. Sleeping or sleep promoting products having been prescribed, the device proposes in a way adapted to the profile of the past day and that of the day to come the use of one type of molecule or another (phytotherapeutic complexes, melatonin, doxylamine, zolpidem, zopiclone…) and manages the duration of treatment and the doses used, taking into account the risks of adverse effects and the development of an addiction. The severity of Bob’s depressive state (30 on Hamilton scale) also justifies the use of an adapted pharmacological treatment [6] in the form of an Selective serotonin reuptake inhibitors (SSRI). A specific follow-up of the health parameters theoretically affected by this treatment is set up in the system allowing an objectification of both its potential therapeutic and undesirable effects.

The device also includes the data collection modules used from the first session, thus allowing Bob a longitudinal follow-up and a better knowledge of his own functioning. This “technologically” increased reflexivity is likely by itself to have effects on the improvement of functioning and symptoms [68]. There are also psycho-educational elements on mental health management (e.g., MyCompass [69]), which can be communicated in direct relation to the context and events, thus improving their relevance and effective integration in practice. In order to avoid the frequently observed decrease in the rate of device use and compliance [70,71], the proposed EMA/EMI applications are designed with particular attention to user commitment and motivation, in particular through the use of gamification [72]. The ergonomics and graphics of the interface allowing interaction with the system are designed to promote a pleasant navigation experience for both patients and clinicians. Overall, the device technologically supports new possibilities of empowerment, self-management and empowerment for Bob, in particular through the understanding of the fine mechanisms that generate and maintain problematic functioning and the operational possibility given to the patient to act on them [48].

The individual data collected by the device are also used to create a data warehouse that serves as a basis for collective analyses of multiple patients. Each element of this database is a localized observation or intervention data collected at time t in a patient p. Tuple analysis {d, p, t} allows both longitudinal intra-individual analyses and inter-individual comparisons or aggregated group analyses, and allows the progressive constitution of a nomothetic and idiographic knowledge base accessible to clinicians and patients to evaluate the effects in real situations of the therapeutic actions implemented.

## 10. Conclusions

The use of connected mobile technologies in the field of mental health has developed particularly rapidly in recent years. Although many applications and connected objects continue to be offered directly by companies, or even individual developers, without their reliability, effectiveness or undesirable effects being scientifically evaluated, it is nevertheless clear that the “technological gadget” stage has now largely passed. Numerous observational and experimental studies of m-health devices have indeed already been conducted, and show statistically and clinically significant effect sizes on many mental disorders and mental health problems.

The central argument of this article is that in order to make the most of these technological innovations, epistemological innovations in the design of mental disorders and therapies are indispensable. The approaches to disorders that we currently use, based on categorical definitions that hypothesize latent causes, were designed with the observational and interventional tools that were available when these conceptualizations emerged. If only for strictly logistical reasons (impossibility of monitoring, observing and intervening on patients every second of their lives), these approaches were based on macroscopic conceptualizations of the disorders associated with specific interventions.

New connected mobile technologies offer the possibility of ubiquitous micro-observations and micro-interventions. This technological finesse of observation and intervention must be matched by an equivalent finesse in models of understanding of disorders and treatments. Our hypothesis is that “symptom network” approaches, such as the one proposed by Borsboom [3] and many other researchers following him open up a particularly interesting avenue for this.

Research and developments on the synergies between new technological approaches to m-health and new epistemological approaches to mental disorders, while promising, remain at an embryonic stage. To enable them to develop effectively, it is also essential to add methodological innovations to them, particularly with regard to effectiveness evaluation mechanisms [73]. The current gold standard of the randomized controlled trial [74] forces experimental studies to limit themselves to evaluating the effect of a single intervention on a single disease entity when, as we have shown, the future lies much more in the possibility of coordinating multiple fine interventions on multiple fine mechanisms.

It is therefore necessary to coordinate efforts now in technological, epistemological and methodological innovations to enable implementation of real-time assessments that have the potential to guide clinical decision toward more appropriate and targeted therapeutic interventions tailored to each individual case, as well as ensuring the rigorous monitoring of standard treatment strategies classically proposed in routine clinical practice thereby improving overall effectiveness and adherence, and thus changing relapsing course and poor prognosis of mental disorders.
